# Outbreak of Murine Infection with *Clostridium difficile* Associated with the Administration of a Pre- and Perinatal Methyl Donor Diet

**DOI:** 10.1128/mSphereDirect.00138-19

**Published:** 2019-03-20

**Authors:** Theresa Mau, Samantha S. Eckley, Ingrid L. Bergin, Katie Saund, Jason S. Villano, Kimberly C. Vendrov, Evan S. Snitkin, Vincent B. Young, Raymond Yung

**Affiliations:** a Division of Geriatric and Palliative Medicine, Department of Internal Medicine, University of Michigan, Ann Arbor, Michigan, USA; b Graduate Program in Immunology, University of Michigan, Ann Arbor, Michigan, USA; c Unit for Laboratory Animal Medicine, University of Michigan, Ann Arbor, Michigan, USA; d In-Vivo Animal Core, University of Michigan, Ann Arbor, Michigan, USA; e Department of Microbiology and Immunology, University of Michigan, Ann Arbor, Michigan, USA; f Division of Infectious Diseases, Department of Internal Medicine, University of Michigan, Ann Arbor, Michigan, USA; g Geriatric Research, Education, and Clinical Care Center, VA Ann Arbor Health System, Ann Arbor, Michigan, USA; University of Wisconsin—Madison; University of South Florida; University of Missouri

**Keywords:** *Clostridium difficile*, mouse, outbreak, veterinary epidemiology

## Abstract

Between October 2016 and June 2017, a C57BL/6J mouse colony that was undergoing a pre- and perinatal methyl donor supplementation diet intervention to study the impact of parental nutrition on offspring susceptibility to disease was found to suffer from an epizootic of unexpected deaths. Necropsy revealed the presence of severe colitis, and further investigation linked these outbreak deaths to a Clostridium difficile strain of ribotype 027 that we term 16N203. C. difficile infection (CDI) is associated with antibiotic use in humans. Current murine models of CDI rely on antibiotic pretreatment to establish clinical phenotypes. In this report, the C. difficile outbreak occurs in F1 mice linked to alterations in the parental diet. The diagnosis of CDI in the affected mice was confirmed by cecal/colonic histopathology, the presence of C. difficile bacteria in fecal/colonic culture, and detection of C. difficile toxins. F1 mice from parents fed the methyl supplementation diet also had significantly reduced survival (*P* < 0.0001) compared with F1 mice from parents fed the control diet. When we tested the 16N203 outbreak strain in an established mouse model of antibiotic-induced CDI, we confirmed that this strain is pathogenic. Our serendipitous observations from this spontaneous outbreak of C. difficile in association with a pre- and perinatal methyl donor diet suggest the important role that diet may play in host defense and CDI risk factors.

**IMPORTANCE** Clostridium difficile infection (CDI) has become the leading cause of infectious diarrhea in hospitals worldwide, owing its preeminence to the emergence of hyperendemic strains, such as ribotype 027 (RT027). A major CDI risk factor is antibiotic exposure, which alters gut microbiota, resulting in the loss of colonization resistance. Current murine models of CDI also depend on pretreatment of animals with antibiotics to establish disease. The outbreak that we report here is unique in that the CDI occurred in mice with no antibiotic exposure and is associated with a pre- and perinatal methyl supplementation donor diet intervention study. Our investigation subsequently reveals that the outbreak strain that we term 16N203 is an RT027 strain, and this isolated strain is also pathogenic in an established murine model of CDI (with antibiotics). Our report of this spontaneous outbreak offers additional insight into the importance of environmental factors, such as diet, and CDI susceptibility.

## Abstract (precis)

Clostridium difficile infection (CDI) has become the leading cause of infectious diarrhea in hospitals worldwide, owing its preeminence to the emergence of hyperendemic strains, such as ribotype 027 (RT027). A major CDI risk factor is antibiotic exposure, which alters gut microbiota, resulting in the loss of colonization resistance. Current murine models of CDI also depend on pretreatment of animals with antibiotics to establish disease. The outbreak that we report here is unique in that the CDI occurred in mice with no antibiotic exposure and is associated with a pre- and perinatal methyl supplementation donor diet intervention study. Our investigation subsequently reveals that the outbreak strain that we term 16N203 is an RT027 strain, and this isolated strain is also pathogenic in an established murine model of CDI (with antibiotics). Our report of this spontaneous outbreak offers additional insight into the importance of environmental factors, such as diet, and CDI susceptibility.

## INTRODUCTION

Clostridium difficile is a spore-forming, Gram-positive obligate anaerobe that has become the leading cause of infectious diarrhea in hospitals worldwide. On a yearly basis, nearly half a million cases of Clostridium difficile infection (CDI) are reported in the United States, with an approximated 29,000 CDI-related deaths (1). Exposure to C. difficile can have varied outcomes ranging from asymptomatic intestinal colonization to severe diarrhea, development of pseudomembranous colitis, and death (2). CDI risk is associated with disruption of the gut microbiota, for example, following antibiotic administration (2–4), that leads to a loss of resistance toward C. difficile colonization.

Following spore germination and establishment of the vegetative form of C. difficile, disease results from the production of the large clostridial toxins TcdA and TcdB (5). These enterotoxins inactivate small GTPases (6–9) (e.g., Rho, Rac, Cdc42) and lead to the rearrangement of the actin cytoskeleton of intestinal epithelial cells. Consequently, mucosal epithelium damage ensues from cellular apoptosis (10) and the induction of colitis via toxin-mediated inflammation. In humans, the increasing clinical presence of C. difficile epidemic strains in the United States and West Europe (11) is attributed to antibiotic use and the rise of endemic strains, such as NAP1/027/BI. Ribotype 027 (RT027) strains produce higher quantities of TcdA and TcdB (12) and express an additional C. difficile transferase (CDT) binary toxin encoded by *cdtA* and *cdtB* (13). The binary toxin also disrupts the cytoskeleton and increases bacterial adhesion to intestinal epithelial cells (14). The exact role of CDT in C. difficile virulence remains undefined, but it has been suggested that CDT plays a significant role in worsened clinical patient outcomes (15) and increases of CDI recurrence (16).

Mouse models have been developed to study the pathogenesis of CDI. These models generally require administration of antibiotics to disrupt the microbiota prior to C. difficile exposure. A number of antibiotic regimens have been employed to render animals susceptible to CDI (17–19). The importance of the indigenous microbiota in mediating colonization resistance against CDI is highlighted by the fact that germfree animals are inherently sensitive to colonization and disease when exposed to C. difficile (20).

Here, we report a spontaneous outbreak of C. difficile due to a strain of RT027 that occurred in a mouse colony that was associated with the administration of a specific pre- and perinatal diet (in the original study, a methyl supplementation donor diet [21–24] was administered to F0 mice, and we study diet-induced obesity in F1 mice). Given that CDI mouse models typically rely on prior gut microbiota disruption via antibiotics to establish disease (25), this outbreak is unique in that it occurred in the absence of antibiotic use and, instead, in association with an altered diet.

## RESULTS

### Recognition of an outbreak of severe typhlocolitis in a cohort of mice undergoing pre- and perinatal diet manipulation.

Typhlocolitis was observed in a cohort of F1 mice from a study involving parental (F0) pre- and perinatal methyl donor supplementation (MS) and postweaning intake of normal diet (ND) or 42% high-fat diet (HFD) chow. The purpose of the original study was to evaluate MS diet epigenetic effects on obesity development in later life and at various life stages of the F1 mice.

Over the course of 254 days from 4 October 2016 to 15 June 2017, 57 of 207 mice in the MS diet study were found dead with no premonitory signs (*n* = 39) or were euthanized for moribund conditions (*n* = 18), including hunched posture, lethargy, dyspnea, and mucoid stool with perineal staining. Severe necrotizing typhlocolitis with pseudomembrane formation and severe submucosal edema were observed at necropsy in early cases and were suggestive of C. difficile colitis (25). This led to screening of affected animals for C. difficile, which was subsequently identified in a number of these mice, suggesting a potential outbreak. To facilitate investigation of this outbreak, we developed the following case definition: confirmed cases (i) had presentation of a compatible clinical syndrome and either (ii) had histopathologic cecal/colonic lesions consistent with C. difficile infection or (iii) tested positive for C. difficile bacteria or C. difficile toxin. Of the 57 animals that died during the study period, we were able to complete analysis on 36 mice and determined that 25 of these mice fit our case definition. We found that 12 mice had causes of death unrelated to the outbreak, and 20 mice had no tissues available for examination (Table 1).

**TABLE 1:** Enumeration of mice affected by the outbreak, including confirmed and excluded deaths

Mouse group^*a*^	Total no. of mice	No. of dead mice	No. of examined mice that met the case definition^*b*^	No. of examined mice that did not meet the case definition	No. of mice with no tissue available
All F0 mice^*c*^	24	1	0	1	0
All F1 mice	183	56	25	11	20
Control-diet (fed to F0) F1 mice	97	15	3	8	4
MS diet (fed to F0) F1 mice	86	41	22	3	16
Normal-chow (fed to F1) F1 mice	118	38	22	5	11
HFD chow (fed to F1) F1 mice	65	18	3	6	9

a MS, methyl donor supplementation; HFD, high-fat diet.

b Mice met the case definition either by their histopathology results or the presence of C. difficile or C. difficile toxins.

c F0 mice were not included in the survival curve analysis.

**FIG 1:**
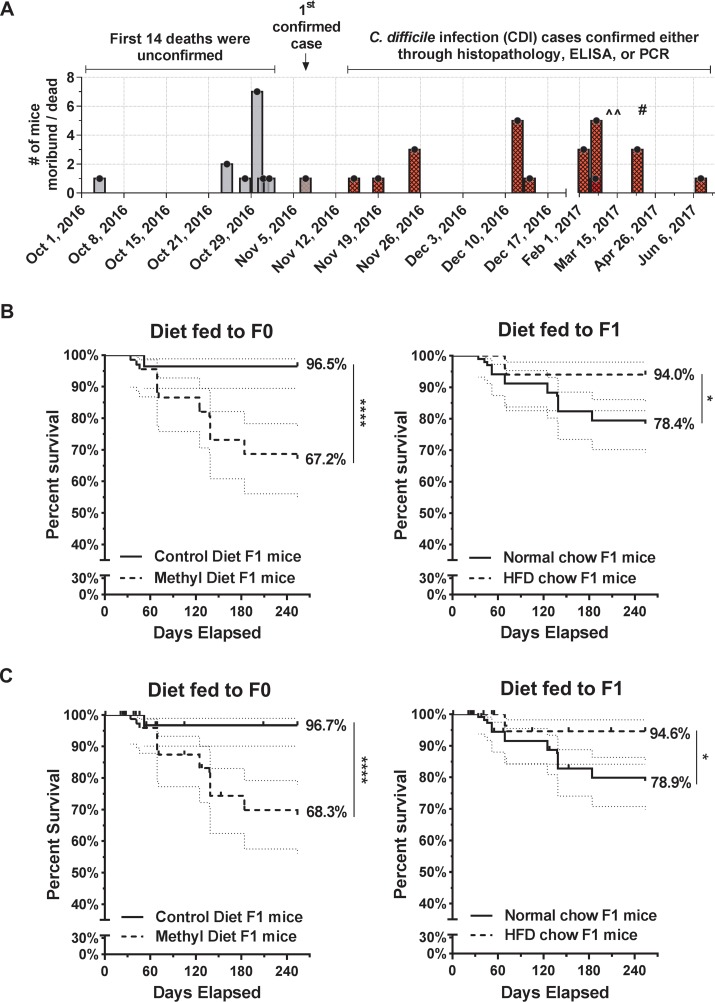
Methyl donor diet mice had higher total mortality during the outbreak. (A) Epidemic curve depicting the first set of unconfirmed cases that triggered the outbreak investigation and the subsequent cases determined to be consistent with C. difficile infection. ^^ denotes environmental swab tests on 9 March 2017, 15 March 2017, and 24 October 2017 (not shown). # denotes plenum plus fecal pellet testing on 14 April 2017. (B) Kaplan-Meier survival curves of mouse colonies comparing the percentages of survival of the F1 mice on the pre-/perinatal control diet (*n* = 85, excluding 8 for not meeting the case definition and 4 for unavailable tissues) versus those on the pre-/perinatal methyl supplementation diet (*n* = 66, excluding 3 for not meeting the case definition and 16 for unavailable tissues) and of F1 mice on the normal-chow diet (*n* = 101, excluding 5 for not meeting the case definition and 11 for unavailable tissues) versus 42% on the high-fat diet (*n* = 50, excluding 6 for not meeting the case definition and 9 for unavailable tissues) over the course of 8.5 months. Ninety-five percent confidence intervals are graphed (light dashed lines). (C) Kaplan-Meier survival curves including all F1 mice (*n* = 183) in analyses but entering previously excluded mice as “0” censored. Survival curves compare control-diet (fed to F0) F1 mice (*n* = 97) to MS diet (fed to F0) F1 mice (*n* = 86). A second curve compares normal chow (fed to F1) F1 mice (*n* = 118) to 42% HFD chow (fed to F1) F1 mice (*n* = 65). *, *P* < 0.05; ****, *P* < 0.0001, Mantel-Cox log-rank test.

We constructed an epidemic curve to follow the course of this outbreak. The initial 14 deaths in the colony that led to the outbreak investigation were included in this epidemic curve (Fig. 1A), but as most of these animals were found dead or their intestinal tissue was not examined, case definitions could not be assigned to these mice. Over the course of the outbreak study, there were 86 MS diet and 97 control diet F1 mice. We excluded all F0 mice and F1 mice that had no tissue available or were examined but did not meet case definition. Kaplan-Meier survival curve analysis on only the confirmed cases of CDI shows lower survival of the MS diet mice (*n* = 66) compared to control diet mice (*n* = 85) (Fig. 1B). Curve analysis indicates that on day 254, which is the day on which the last outbreak case was identified, 96.5% of the control mice survived but that only 66.7% of the MS mice remained (*P* < 0.0001). A curve comparison of the confirmed CDI cases based on F1 diets—the HFD (*n* = 50) versus the ND (*n* = 101) group—suggests that HFD-fed mice had higher survival percentages (*P* < 0.02) (Fig. 1B); however, there were 65 F1 mice on the HFD, while 118 F1 mice were on the ND in the initial colony (Table 1). For comparison, we also included a Kaplan-Meier survival curve analysis on all F1 mice, incorporating them as censored (lost) for analyses (Fig. 1C).

While attempting to control this outbreak, breeders on the MS diet and control diet were transferred to a separate room in January 2017 based on negative fecal enzyme-linked immunosorbent assay (ELISA) results. The remainder of the colony was ultimately transferred to containment housing. On 14 April 2017, PCR of pooled plenum and fecal pellets revealed that 8/8 rows on one rack and 1/2 rows on a second rack were positive for C. difficile. Environmental samples on 3 separate dates (9 March 2017, 15 March 2017, and 24 October 2017) tested negative for C. difficile. The timeline of the plenum swabs, fecal sampling, and environmental tests is also included on the epidemic curve (Fig. 1A).

### Identification of C. difficile in animals with typhlocolitis.

**FIG 2:**
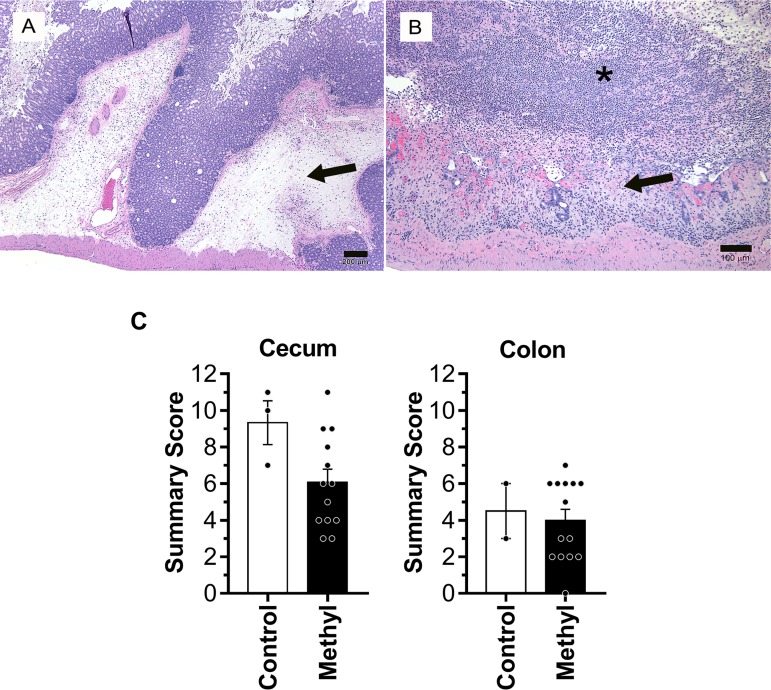
Necropsies and histopathology indicate typhlocolitis as the primary diagnosis of affected animals. (A) Representative histology of mouse cecum in a C. difficile culture-positive case exhibiting severe submucosal edema and inflammation (arrow). Hematoxylin and eosin stain. Bar = 200 µm. (B) Representative histology of an area of cecum in the same case displaying mucosal necrosis (arrow) and extensive pseudomembrane formation (*), consisting of neutrophils and sloughed enterocytes in a fibrinous matrix. Hematoxylin and eosin stain. Bar = 100 µm. (C) Histological severity scores for the 17 mice showing typhlocolitis, out of 24 clinically affected mice for which tissues were available. Severity was scored by a board-certified veterinary pathologist using an established murine C. difficile scoring system. Each category, edema, inflammation, or epithelial damage, was individually scored 0 to 4, and a summary score was generated (range, 0 to 12). Of the 17 mice, 1 mouse had no cecum score and 1 mouse had no colon score due to tissue autolysis of those samples. Therefore, for cecum scores, there were 3 control-diet F1 mice and 13 methyl supplementation (MS) diet F1 mice. For colon scores, 2 mice were on the control diet and 14 mice were on the MS diet.

Gastrointestinal (cecum and colon) tissues were available for histologic evaluation in 24 of the total 57 animals found dead or euthanized during the outbreak (Fig. 2). Seventeen of 24 animals were found to have necrotizing and pseudomembranous typhlocolitis consistent with CDI, as seen in experimental murine models (25). Histological findings included striking submucosal edema of the cecum and colon, accompanied by marked neutrophilic inflammation and necrosis and sloughing of the superficial mucosa and midportions of the mucosa, often with pseudomembrane formation (Fig. 2A and B). We were also able to collect and submit fecal and/or cecum and colonic samples for testing from 14 of the 17 animals with histologically evident typhlocolitis. Twelve of the 14 animals tested positive for C. difficile TcdA and TcdB via ELISA, and 2 tested positive for C. difficile bacteria via PCR. Fourteen were F1 mice from the MS diet group, and 3 were from the control diet group (Fig. 2C). The cecum summary score (Fig. 2C) of mice on the MS diet (*n* = 13) was 6.1 ± 2.6, and it was 9.3 ± 2.1 for mice on the control diet (*n* = 3). The colon summary scores (Fig. 2C) were 4.0 ± 2.2 for mice on the MS diet (*n* = 14) and 4.5 ± 2.1 for mice on the control diet (*n* = 2). One MS diet F1 mouse had missing colon scores, and one control diet F1 mouse had missing cecum scores due to tissue autolysis. Given that significantly fewer control diet F1 mice (*P* < 0.0001) were affected in the outbreak (Fig. 1B), we collected fewer control samples and, thus, cannot draw conclusions on the effect of the F0 diet on disease severity between the two groups of F1 mice.

Overall, we observed that the histology score was higher in cecum than in colon in most animals, consistent with experimental murine C. difficile models (25). For the remaining 7 out of 24 histologically evaluated animals that did not have evidence of typhlocolitis, we identified alternate causes of death/morbidity, including tumors (*n* = 2), bacterial enteritis (*n* = 2), or reasons that were undetermined by histology (*n* = 3). Finally, there were 12 out of 36 analyzed animals that had no gastrointestinal tissue available to be histologically evaluated, and 8 of these animals tested positive for C. difficile bacteria (*n* = 2) and C. difficile toxins A and B (*n* = 6), which then allowed us to include these 8 as confirmed cases of C. difficile-related deaths. The 4 animals that tested negative for C. difficile toxin were found to have an alternative cause of death (endometritis [*n* = 1]) or an undetermined cause (*n* = 3), and naturally, these were not included in the confirmed cases of C. difficile.

**FIG 3:**
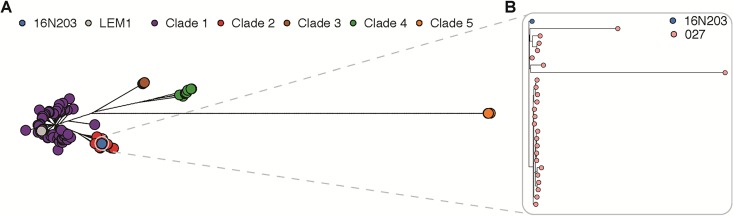
Isolation and genomic sequencing confirm the NAP1/027/BI Clostridium difficile strain. (A) Phylogenetic relationship between the mouse outbreak strain and representative C. difficile isolates. Isolates include publicly available clinical genomes, clinical isolates collected at the University of Michigan, and two mouse strains (16N203 and LEM1). A maximum-likelihood tree was generated from the recombination filtered, polymorphic core genome positions in all isolates (*n* = 438). Clade 1 (purple) includes reference strain CD630 and mouse strain LEM1 (gray); clade 2 (red) includes the epidemic RT027 strain. Clades 1 to 5 are as previously defined (30, 54, 55). (B) The tree in panel A was a subset including only RT027 isolates. The mouse outbreak 16N203 strain (blue) clusters with the RT027 isolates (pink), an RT in clade 2 (red).

Isolation of C. difficile was performed by the use of selective culture. A C. difficile strain (designated 16N203 for the animal from which it was initially isolated) was obtained in pure culture. A PCR (26) confirmed that C. difficile bacteria were present in fecal samples (see Fig. S1A in the supplemental material), and this strain was also positive for the toxin genes *tcdA* and *tcdB* and *cdtA* and *cdtB* by PCR (27) (Fig. S1B). Whole-genome sequencing of strain 16N203 was undertaken, and a whole-genome phylogeny, including of a representative set of previously sequenced C. difficile isolates, was constructed (Fig. 3A). These isolates contain publicly available clinical genomes (28–30), clinical isolates collected at the University of Michigan, and two mouse strains (16N203 and LEM1). LEM1 is an indigenous murine spore-forming C. difficile strain identified and isolated from mice acquired from common mouse vendors, Jackson Laboratories and Charles River Laboratories (31). LEM1 appears to not be highly virulent and can protect against the closely related but more virulent strain VPI 10463, at least in mice with the C57BL/6J or BALB/c background (31). In our data, the murine strain LEM1 clusters with clade 1, which contains reference strain CD630, while the outbreak strain fell within the diversity of clade 2, which contains RT027 isolates (Fig. 3A). When a phylogenetic tree was constructed with only RT027 isolates, the outbreak 16N203 strain clustered with isolates derived from human patients with clinical CDI (32) (Fig. 3B).

10.1128/mSphereDirect.00138-19.1 FIG S1 PCR identification of Clostridium difficile and its associated toxins (*tcdA*, *tcdB*, and the binary *cdtA* and *cdtB* toxin). (A) PCR (26) confirming C. difficile-specific identification. Lane 1, 100-bp ladder; lane 2, 16N203-1; lane 3, 16N203-2; lane 4, 16N203-3; lane 5, 16N203-4; lane 6, C. difficile 630; lane 7, water. (B**)** PCR (27) confirming the presence of CDI toxins: toxin A (*tcdA*), toxin B (*tcdB*), and the binary toxin (*cdtA* and *cdtB*). Lane 1, 100-bp ladder; lane 2, 16N203-1; lane 3, 16N203-1; lane 4, 16N203-2; lane 5, 16N203-3; lane 6, 16N203-4; lane 7, C. difficile R20291; lane 8, C. difficile 630. Download FIG S1, TIF file, 0.8 MB. Copyright © 2019 Mau et al. 2019 Mau et al. This content is distributed under the terms of the Creative Commons Attribution 4.0 International license.

### C. difficile strain 16N203 is fully virulent in a mouse model of antibiotic-induced CDI.

**FIG 4:**
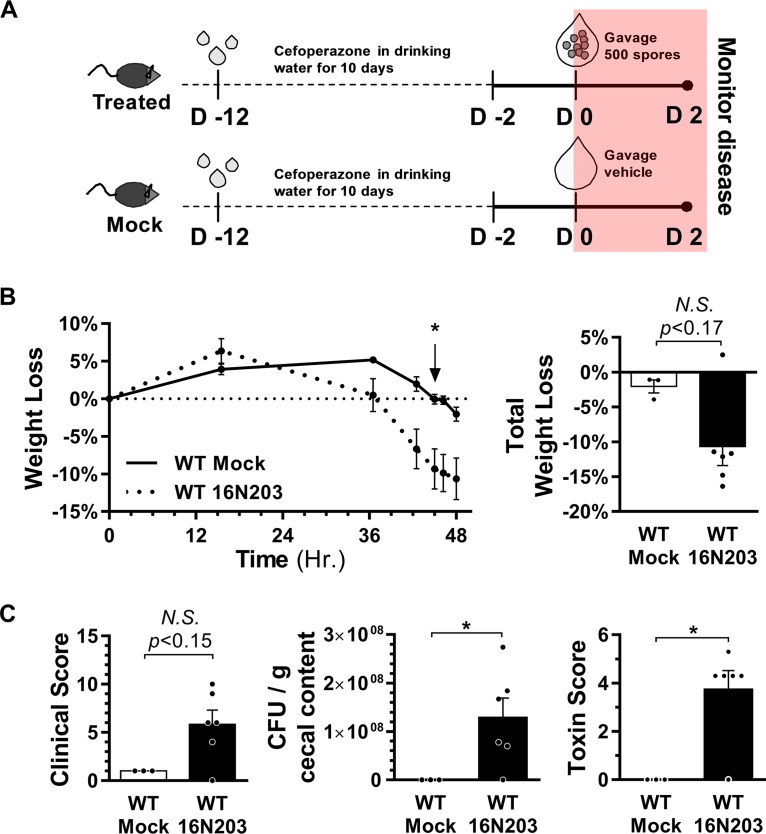
The C. difficile outbreak strain induces CDI in a standard mouse model with antibiotics. (A) CDI mouse model with cefoperazone in drinking water used to assess the virulence of the outbreak 16N203 strain. Wild-type (WT) mice were administered cefoperazone in drinking water for 10 days on day −12 (D −12) and then orally gavaged with either 16N203 spores (*n* = 6) or the vehicle (distilled water for mock [*n* = 3] controls) on day 0 (D 0). Mice were monitored for disease between days 0 and 2. (B) Body weight loss over the course of 48 h. The total percentage of body weight loss is shown in column graph. (C) Clinical score, colonization, and toxin scores at 48 h. For weight loss, clinical, colonization, and toxin scores, the Mann-Whitney test was used for statistical analyses. *, *P* < 0.05. *N.S.*, not significant.

We had previously demonstrated that C. difficile strains have variable virulence in an established mouse model where CDI susceptibility is conveyed by treatment with the antibiotic cefoperazone (25). To determine whether the pathogenicity of the outbreak strain 16N203 was unique to the dietary model, we assessed whether the outbreak strain 16N203 was virulent in this established model of CDI (Fig. 4A). C57BL/6J mice on a standard diet and without previous dietary manipulations were treated with cefoperazone in drinking water for 10 days and then switched back to plain water. Two days after antibiotics were stopped, mice were challenged with either 500 spores of the 16N203 outbreak strain or vehicle via oral gavage. Mice challenged with 16N203 spores during the 48-h study time course exhibited significant weight loss (*P* < 0.05), while mock-treated mice maintained weight (Fig. 4B). Mice challenged with C. difficile 16N203 had higher levels of cecal colonization (*P* < 0.03), with detectable toxin in intestinal contents (*P* < 0.05), and developed clinical signs compatible with severe CDI (Fig. 4C).

## DISCUSSION

In this report, we describe an outbreak of C. difficile infection (CDI) and colitis in laboratory mice associated with diet manipulation. This outbreak is interesting because spontaneous, symptomatic CDI is unusual in laboratory mice, and symptomatic colitis due to experimental CDI generally requires pretreatment with antibiotics (31, 33). Subclinical colonization of mice has been reported previously. In one study, it was noted that mice could be colonized with C. difficile with or without prior antibiotic treatment, but this colonization was at a very low level and not associated with any clinical disease (19). Administration of the antibiotic clindamycin was associated with increases in the levels of colonization but again without development of clinical colitis. It has been also reported that wild rodents can harbor toxigenic C. difficile strains, again without any overt disease (34). One group reported that laboratory mice obtained from multiple sources appear to harbor LEM1, an indigenous C. difficile strain (31). Colonization with LEM1 in the same study did not result in disease and in fact was associated with protection from disease following challenge with another strain of C. difficile that generally produces severe colitis in experimental models of CDI. In contrast, while LEM1 clusters with clade 1 (containing reference strain CD630 [RT12]), the outbreak strain in our study clustered in clade 2 with patient-derived RT027 isolates and was associated with clinical disease in both spontaneously infected and experimentally infected mice. The source of this strain remains unclear, and our attempt at revealing a source with environmental sampling did not yield conclusive results. Ribotyping and genetic analyses suggest that a potential source was through fomites or human carriers, but the source may have been gone by the time of environmental investigation.

The mechanism underlying the unexpected increased susceptibility of these mice is also not entirely clear. Small-animal models to recapitulate CDI pathogenesis generally rely on antibiotic pretreatment prior to experimental challenge with C. difficile, even with virulent strains (33). As noted, the current outbreak is unusual in that there were no antibiotics administered to the animals; instead, susceptibility to colonization and colitis was associated with dietary (peri- and prenatal) manipulation. Our current understanding of the pathogenesis of CDI attributes the loss of colonization resistance to the altered structure and function of the indigenous intestinal microbiota (35). However, given the unexpected nature of the outbreak, we were not positioned to determine if there were changes in the microbiota associated with the increased susceptibility that we observed in MS F1 mice. Antibiotics, which can cause widespread changes in the indigenous microbiota, are associated with the development of clinical CDI in humans, but other factors that alter microbial populations may also convey susceptibility.

In our study, increased C. difficile-associated deaths were seen in the offspring of mice receiving the methyl supplementation (MS) diet. Diet may be a factor that can alter the community structure and function of the intestinal microbiota, and diet-related susceptibility to CDI has been evaluated in some studies (36, 37). Patients receiving enteral tube feeding had increased risk of developing CDI (38). Tube feeds given in the form of an elemental diet (i.e., a diet that is entirely absorbed within the small bowel) are thought to deprive the colonic bacteria of nutrition in the form of fiber and resistant starch, increasing the risk of CDI (39). Microbiota-accessible starches may protect against CDI, as in murine studies where feeding such carbohydrates can suppress experimental CDI (40). This protection was associated with an increase in short-chain fatty acids, which are the metabolic products of microbial fermentation of nondigestible carbohydrates. Another study showed that a low-protein diet (which had reciprocal increases in carbohydrate composition) could be protective in a mouse model of CDI (41). It was recently demonstrated that zinc deficiency, in addition to macronutrients, could alter the microbiota and increase susceptibility to experimental CDI (42).

It should be noted that, unlike with our study, the diet-related susceptibility or amelioration of experimental CDI discussed above occurred in models where the microbiota was still altered via antibiotic administration. In an earlier study of hamsters fed an atherogenic diet in the absence of antibiotic administration, unexpected diarrhea and death due to colitis were observed in animals, starting 45 days after diet manipulation (43). As in our study, toxigenic C. difficile was isolated from affected animals, although typing of the strain was not performed. It is interesting to note that in these hamsters, the atherogenic diet increased their susceptibility to CDI, but in our study, F1 mice that were on a 42% high-fat diet (HFD) exhibited increased survival, though it should be noted that this observation was limited by the fact that there were significantly more normal-diet mice than HFD mice in the mouse colony at the time.

While hamsters are exquisitely susceptible to C. difficile, symptomatic murine C. difficile colitis outbreaks in the absence of antibiotic administration are very rare. There is precedence for disease outbreaks in mice associated with C. difficile, but the affected mice have had significant immune alterations that increased their susceptibility (44, 45). One such outbreak occurred in a murine experimental autoimmune encephalomyelitis model generated via the administration of pertussis toxin and a myelin oligodendrocyte glycoprotein fragment (45). Those authors speculated that the stress of this experimental manipulation or the development of the autoimmune disease altered the microbiota in a manner that leads to susceptibility to CDI, but they did not profile the microbiota in these animals. Although we were unable to characterize the microbiota in our study due to the unexpected nature of the outbreak, a future avenue to explore is diet-associated microbial community alterations that may convey susceptibility.

The specific mechanisms by which our methyl donor diet alters susceptibility to CDI are unclear. A similar methyl supplementation maternal diet was shown to increase colitis risk in a chemically induced inflammatory bowel disease (IBD) model using dextran sulfate sodium (DSS) (46, 47). F1 mice from methyl-fed dams had worsened colitis and significantly higher mortality than control F1 mice (46). Colonic mucosal bacterial diversity analyses indicated striking composition variation, including significantly higher prevalence of *Clostridia* in methyl diet F1 mice. In addition, there was an increase in numbers of *Firmicutes* (*Lachnospira*, *Oscillospira*, *Ruminococcus*, and *Catonella*) and a decrease in numbers of *Parabacteroides* and *Eubacterium* organisms in methyl F1 mice compared to numbers in controls. This suggests that dysbiosis between colitogenic (*Firmicutes*) and anti-inflammatory bacteria (*Parabacteroides*) following prenatal methyl supplementation may have led to the colitis-prone phenotype (46). A fecal-microbiome transfer (FMT) via cage swapping (between methyl F1 mice and germfree mice) was sufficient to worsen DSS-induced colitis in germfree mice, indicating that the maternal methyl donor diet alone may enhance a colitis-prone microbiota profile. While this study cannot be directly compared to C. difficile colitis, it strongly suggests that a prenatal methyl donor diet is sufficient to generate a procolitic F1 microbiota. In the context of CDI, it remains to be determined whether susceptibility is due strictly to altered community composition, creating a niche for C. difficile colonization, or whether microbial changes directly promote colitis-enhancing inflammatory responses. While unexpected, the current spontaneous outbreak of CDI in the absence of antibiotics in mice with parents fed an altered pre- and perinatal diet may ultimately provide additional insight into the interplay of diet, host responses, and the microbiota that mediates CDI and colitis susceptibility.

## MATERIALS AND METHODS

### Animals.

The animal care and use program at the University of Michigan is AAALAC accredited. All procedures involving the animals and their care were approved by the University of Michigan Institutional Animal Care and Use Committee. Mice were housed in autoclaved positive-pressure positive/negative-control individually ventilated cages (P/NV IVC; Allentown, Allentown, NJ) with corncob bedding (The Andersons, Frontier) and provided with reverse-osmosis-deionized (RO/DI) water through automated water systems (Edstrom, Waterford, WI). Animal-housing rooms were maintained on a cycle of 12 h of light/12 h of dark, with relative humidity at 30 to 70% and a temperature of 72 ± 2°F (22.2 ± 1.1°C). For specific-pathogen-free (SPF) rooms, cage changing and experimental procedures were performed under a laminar-flow cage change station (AniGARD VF; Baker Company, Sanford, ME) or in laminar-flow benches, using a cold sterilant (Spor-Klenz; Steris, St. Louis, MO) for disinfecting gloved hands or transfer forceps. For biocontainment rooms (animal biosafety level 2), procedures were performed under a biosafety cabinet (SterilGARD; Baker Company, Sanford, ME) using a sporicidal disinfectant cleaner (Perisept; Triple S catalog number 48027). The health surveillance program for SPF colonies included quarterly testing of dedicated soiled-bedding sentinel animals via fecal and perianal swab PCR or serology and PCR of exhaust plenum swabs for fur mites. Health surveillance results indicated that the mice were negative for mouse rotavirus, mouse hepatitis virus, minute virus of mice, ectromelia virus, Theiler mouse encephalomyelitis virus, lymphocytic choriomeningitis virus, mouse adenovirus, mouse parvovirus, mouse polyomavirus, pneumonia virus of mice, reovirus, Sendai virus, Mycoplasma pulmonis, pinworms (*Syphacia* spp. and *Aspiculuris* spp.), and fur mites (*Myobia musculi*, *Myocoptes musculinus*, and *Radfordia affinis*).

### Dietary study.

In the initial dietary study, young-adult (8-wk) C57BL/6J mice were purchased from Jackson Laboratories. All custom diets prefaced with “TD” are produced and distributed by Harlan-Teklad (Madison, WI). Mice were acclimated for 2 weeks before being fed either the control (TD.06689) or a methyl donor supplementation (MS) diet (TD.110144). The MS diet contained 12 g methionine per kg of body weight, 16.5 g/kg choline, 15 g/kg betaine, 16.5 mg/kg folic acid, 1.56 mg/kg vitamin B_12_, and 200 mg/kg Zn. Two weeks after starting the diet, female and male mice were paired for mating. Mated F0 mice were fed the diet throughout pregnancy and lactation. F1 mice were weaned at 28 days. The F1 mice were then placed on a standard-chow diet (PicoLab laboratory rodent diet 5L0D; LabDiet, St. Louis, MO) or a 42% high-fat diet (HFD) (TD.88137).

### Outbreak necropsy and assessment.

Dead or clinically affected animals were detected during routine daily health checks. Veterinary staff assessed for any clinical sign of gastrointestinal or systemic disease, such as diarrhea, lethargy, or unkempt appearance, and euthanized animals via CO_2_ inhalation followed by induction of the bilateral pneumothorax. Complete necropsy was performed, with select tissues processed for histologic examination.

Fecal PCR of selected mice in the colony was performed by a commercial laboratory (Charles River Laboratories, Wilmington, MA) to confirm SPF status. Fecal and/or cecum and colonic samples were submitted for ELISA for C. perfringens type A toxin and C. difficile toxins A and B (Veterinary Diagnostic Laboratory, Michigan State University, Lansing, MI) or for real-time fluorogenic PCR for C. difficile targeting the 23S rRNA gene (Charles River Laboratories, Wilmington, MA). Cecum and colon tissues as well as tissues from other organs were fixed in 10% neutral buffered formalin for a minimum of 24 h and then routinely processed to paraffin, sectioned, and stained with hematoxylin and eosin by the University of Michigan In-Vivo Animal Core (IVAC) histology laboratory. A board-certified veterinary pathologist blind to the diet groups evaluated the tissues descriptively in the initial outbreak and subsequently performed severity scoring of cecal and colonic tissues according to our previously published scoring system for experimentally induced C. difficile-associated typhlitis and colitis (25). In brief, slides were evaluated on a 0-to-4 scale for the individual parameters of edema, inflammation, and epithelial damage, and an overall severity score was generated by summing these parameters (scale, 0 to 12).

To investigate the outbreak source and the extent of contamination, environmental and fecal pellet PCR was performed as described above. Swabs were taken from various areas, including cardboard rolls collected for mouse enrichment and the interior of the respective collection bins and supply and exhaust plenums of four autoclaved racks. Within the ABSL2 room, door knobs, biosafety cabinet used for changing cages, clean lixits, and rack supply ducts connected to the building ventilation system were sampled. Each PCR sample tested for the affected mouse colony comprised of pooled fecal pellets (a fecal pellet collected from a representative mouse in each cage in each rack row) combined with the associated rack row’s exhaust plenum swab.

### C. difficile strain isolation, growth conditions, and colony identification.

C. difficile strain 16N203 was isolated from feces frozen at −80°C after collection from a spontaneously affected animal in the dietary study mouse colony. One and a half fecal pellets were thawed, passed into an anaerobic chamber, and diluted in 200 µl sterile anaerobic phosphate-buffered saline (PBS). Twenty microliters was placed into 3 ml of taurocholate cefoxitin cycloserine fructose broth (TCCFB) and incubated at 37°C overnight. A 10-µl loop of the TCCFB enrichment culture was streaked onto taurocholate cefoxitin cycloserine fructose agar (TCCFA) to get an isolated colony of C. difficile. The isolated colony was used for downstream applications.

For colony identification, a 16N203 C. difficile colony was diluted in 15 µl UltraPure water (Invitrogen catalog number 10977-015), heated to 95°C for 20 min, and then used for colony PCR to determine the identity and toxin type of the isolated organism (26, 27). PCR was performed using the following primers for the C. difficile-specific band: 5′-TTGAGCGATTTACTTCGGTAAAGA-3′ (forward) and 5′-CCATCCTGTACTGGCTCACCT-3′ (reverse), along with the universal 16S primers 515F, 5′-GTGCCAGCMGCCGCGGTAA-3′, and E939R, 5′-CTTGTGCGGGCCCCCGTCAATTC-3′ (26). Toxin-specific PCR followed previously published primers by omitting the 16S rRNA gene primers (27). The PCRs were run in a total volume of 25 μl containing GoTaq Green master mix (Promega catalog number M712), primers, and nuclease-free water.

### C. difficile cytotoxin assay.

A cytotoxicity assay was performed as previously described with the following modifications (25). Briefly, ATCC CCL-81 Vero cells were grown to confluence in Dulbecco modified Eagle medium (Gibco catalog number 11965) supplemented with 10% fetal bovine serum (catalog number 16140) and 1% penicillin-streptomycin (Gibco catalog number 15070). Cells were plated to a density of 10^5^ cells/well. Mouse cecal content was diluted 1:10 in sterile PBS, passed through a 0.22-µm filter, and serially diluted to 10^−6^. Filtered samples were tested in duplicate with a corresponding control to which both antitoxin (Techlab catalog number T5000) and sample were added. A positive control of C. difficile TcdA (List Biologicals catalog number 152C) was used. Samples were incubated overnight at 37°C, and the cytotoxic titer was determined as the reciprocal of the highest dilution that produced 80% cell rounding.

### Genome sequencing, variant identification, and comparative genomics.

A colony of C. difficile 16N203 was cultured 18 h anaerobically at 37°C in 13 ml brain heart infusion broth (BD catalog number 211059) plus 0.01% l-cysteine (Sigma catalog number C6852). The culture was spun at 4,500 × *g* for 12 min, washed one time with sterile PBS, and spun at 4,500 × *g* for 12 min, and the pellet was resuspended in 300 μl of DNeasy UltraClean microbial kit microbead solution (Qiagen catalog number 12224-50). The extracted DNA was then prepared for sequencing on an Illumina MiSeq instrument using the Nextera XT kit and sample-specific barcoding. Library preparation and sequencing were performed at the Center for Microbial Systems at the University of Michigan. The quality of reads was assessed with FastQC (48), and Trimmomatic (49) was used for trimming adapter sequences and low-quality bases. Genome assemblies were performed using Spades (50). Variants were identified by (i) mapping filtered reads to the assembled C. difficile strain 630 reference sequence (GenBank accession number NC_009089.1) using the Burrows-Wheeler short-read aligner (BWA), (ii) discarding PCR duplicates with Picard, and (iii) calling variants with SAMtools and bcftools. Variants were filtered from raw results using GATK’s VariantFiltration (Phred score quality > 100, mapping quality > 50, >10 reads of the supporting variant, consensus quality < 0.025). In addition, a custom python script was used to filter out single-nucleotide variants that were (i) <5 bp in proximity to indels, (ii) <10 bp in proximity to another variant, or (iii) not present in the core genome. Maximum-likelihood trees were constructed using core genome variants among the outbreak strain and a representative set of previously sequenced C. difficile genomes in FastTree (51). Multilocus sequence typing (MLST) predictions were made by subjecting genome assemblies to a BLAST search against the PubMLST database for C. difficile (downloaded on 3 January 2017).

### Experimental C. difficile infection.

Eight-week-old wild-type (WT) C57BL/6J mice (6 males and 3 females) were obtained from an in-house breeding colony that was established originally with animals from Jackson Laboratories (Bar Harbor, ME). Mice were treated for 10 days with 0.5 g/liter cefoperazone (MP Biomedicals catalog number 219969505) in sterile distilled water (Gibco 15230-147) as previously described (25). Briefly, animals were allowed to drink antibiotic amended water *ad libitum* and the antibiotic water was changed every other day. After 10 days, antibiotic water was switched to sterile distilled water. After 2 days on water without antibiotics, 3 male and 3 female mice (total treated, 6) were challenged via oral gavage with 500 spores of C. difficile strain 16N203. Spores (preparation described below) were suspended in 50 µl Gibco sterile distilled water, and 3 male mice (total number mock infected, 3) were treated with 50 µl of Gibco water only. Mice were monitored 16 h postinfection, and feces were collected and plated to confirm spore inoculation. Mice were observed every 3 h for 36 h for clinical signs of disease until clinical signs appeared. Mice were euthanized by CO_2_ inhalation when clinical signs appeared or at the study endpoint. Mice were then evaluated at 48 h after C. difficile infection for weight loss, activity, posture, coat, diarrhea, and nose/eye appearance (52). Animals were euthanized, and cecal contents and tissues from the animals were collected for culture and ELISA.

### C. difficile spore preparation.

16N203 was streaked onto TCCFA, and an isolated colony was incubated overnight anaerobically at 37°C in 2 ml Columbia broth (BD catalog number 294420). The next day, the culture was placed into 40 ml of Clospore (53) and incubated anaerobically at 37°C for 8 days. The culture was spun at 3,200 rpm for 20 min at 4°C. The pellet was washed two times with sterile water (Gibco catalog number 15230-147), once with sterile 0.05% Tween 20 (Fisher catalog number BP337), and then once with sterile water. The final pellet was resuspended in 1 ml sterile water. The spore stock was stored at 4°C in sterile water. Prior to gavage, spores were heated to 65°C for 20 min. Spores were plated on TCCFA to determine doses administered to animals.

### Data availability.

The genome sequence of the 16N203 strain was deposited in the NCBI under BioProject ID PRJNA526195 and BioSample accession no. SAMN11089745.
