# Supplementary figures and images for: Outbreak of Murine Infection with Clostridium difficile Associated with the Administration of a Pre- and Perinatal Methyl Donor Diet
Source: mSphere. 2019 Mar 20;4(2):e00138-19. doi: 10.1128/mSphereDirect.00138-19 (PMC6429045; doi:10.1128/mSphereDirect.00138-19)

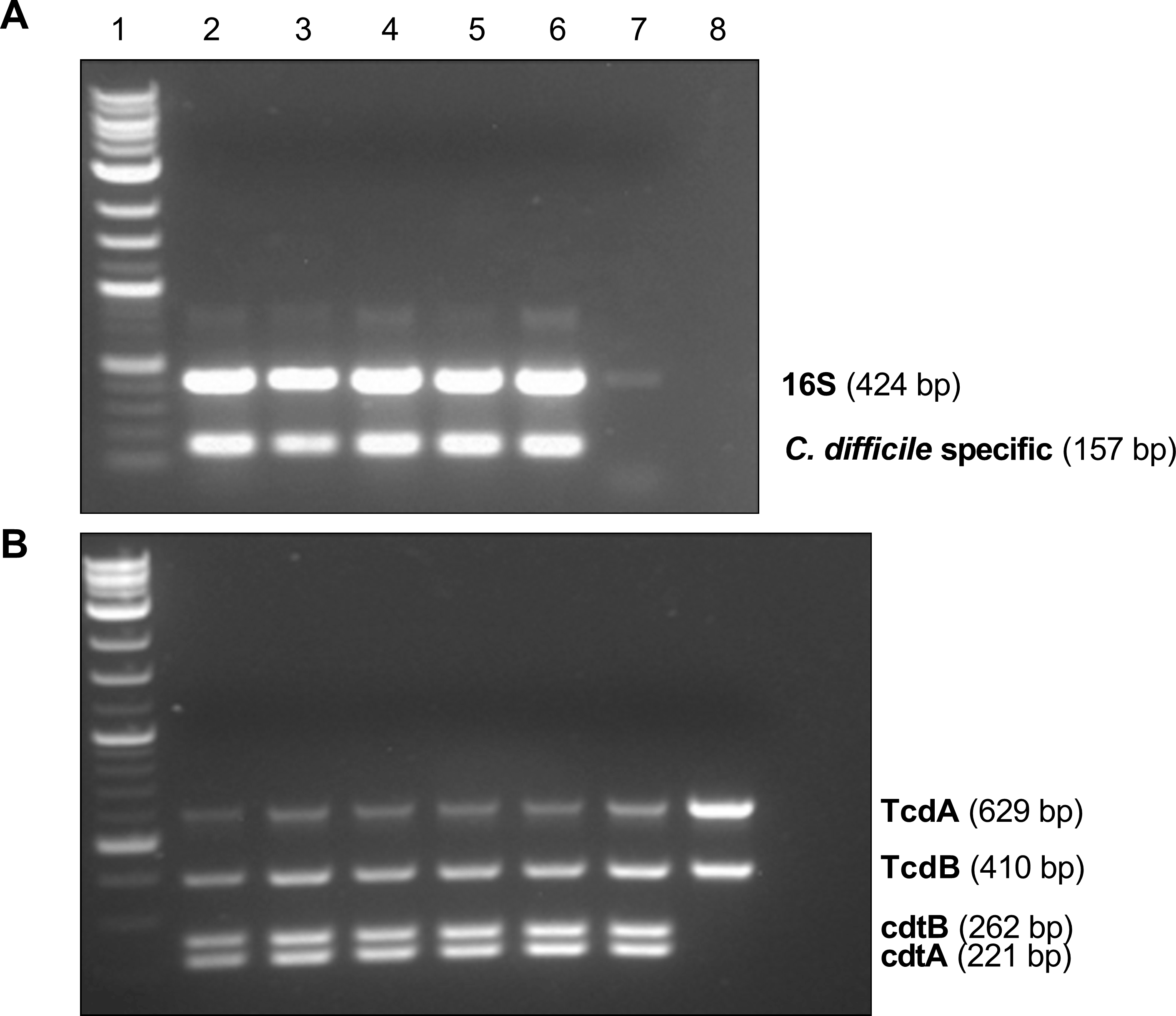

Supplement: FIG S1 [file mSphereDirect.00138-19-sf001.tif]
